# New Insights in Phenothiazinium-Mediated Photodynamic Inactivation of Candida Auris

**DOI:** 10.3390/jof9070717

**Published:** 2023-06-30

**Authors:** Abdênego R. Silva, Fernanda V. Cabral, Camila R. Silva, Daniela F. T. Silva, Anderson Z. Freitas, Adriana Fontes, Martha S. Ribeiro

**Affiliations:** 1 Center for Lasers and Applications, Nuclear and Energy Research Institute (IPEN-CNEN), São Paulo 05508-000, SP, Brazil; abrodrigues@usp.br (A.R.S.); fe_vcabral@hotmail.com (F.V.C.); ramos.camilasilva@gmail.com (C.R.S.); daniela.onn@gmail.com (D.F.T.S.); freitas.az@usp.br (A.Z.F.); 2 Department of Biophysics and Radiobiology, Federal University of Pernambuco, Recife 50670-901, PE, Brazil; adriana.fontes@ufpe.br

**Keywords:** biofilm, 1,9-dimethyl methylene blue (DMMB), lipid peroxidation, methylene blue (MB), mitochondrial membrane potential, oxidative stress, red LED

## Abstract

In recent years, *Candida auris* has emerged as a hazardous hospital-acquired pathogen. Its resistance to antifungal treatments makes it challenging, requiring new approaches to manage it effectively. Herein, we aimed to assess the impact of photodynamic inactivation mediated by methylene blue (MB-PDI) or 1,9-dimethyl MB (DMMB-PDI) combined with a red LED against *C. auris*. To evaluate the photoinactivation of yeasts, we quantified colony-forming units and monitored ROS production. To gain some insights into the differences between MB and DMMB, we assessed lipid peroxidation (LPO) and mitochondrial membrane potential (ΔΨm). After, we verified the effectiveness of DMMB against biofilms by measuring metabolic activity and biomass, and the structures were analyzed through scanning electron microscopy and optical coherence tomography. We also evaluated the cytotoxicity in mammalian cells. DMMB-PDI successfully eradicated *C. auris* yeasts at 3 μM regardless of the light dose. In contrast, MB (100 μM) killed cells only when exposed to the highest dose of light. DMMB-PDI promoted higher ROS, LPO and ΔΨm levels than those of MB. Furthermore, DMMB-PDI was able to inhibit biofilm formation and destroy mature biofilms, with no observed toxicity in fibroblasts. We conclude that DMMB-PDI holds great potential to combat the global threat posed by *C. auris*.

## 1. Introduction

Fungal pathogens are a growing global public health concern, as they are becoming more and more common and resistant to the conventional antifungals currently used in clinical practice. Indeed, invasive fungal diseases are increasing worldwide, mainly for immunocompromised patients such as patients with HIV, chronic lung disease, tuberculosis, cancer and diabetes mellitus [1]. In front of this scenario, the World Health Organization released the first-ever list of health-threatening fungi, which aims to focus on research and development to reinforce the global response to fungal infections and antifungal resistance. In this list, *Candida auris* is prioritized in the critical group [2].

*C. auris* is an emerging pathogen first reported in 2009 by Japanese researchers that has now disseminated globally [3]. *C. auris* strains stand out among other *Candida* spp. for their capacity to disseminate in healthcare facilities and cause severe infections in patients. It is suggested that its ease of spread is due to climate change and anthropic actions, given its thermal and salinity tolerance characteristics [4]. Additionally, this fungal species is resistant to the most widely used antifungals, leading to a high risk of morbidity and mortality [2].

Photodynamic inactivation (PDI) rises in this context as a promising approach to combat *C. auris*. PDI uses the activation of a photosensitizer (PS) by light at a proper wavelength to produce reactive oxygen species (ROS), killing microorganisms via oxidative stress. PDI is an attractive option against *C. auris*, owing to its affordability, effectiveness to inactivate a wide range of pathogenic microorganisms, minimal harm to mammalian cells, inability to select resistant strains and capability of reducing virulence factors in *Candida* spp. [5,6,7].

Some PSs have already been reported to be effective in mediating PDI against *C. auris*, such as phenothiazines, phthalocyanines and hypocrellin [8,9,10]. Particularly, among phenothiazines, methylene blue (MB) has been widely employed against several pathogens. MB is a hydrophilic dye that binds to negatively charged components of the cells. It can absorb red light, produce high levels of ROS and exhibit no toxicity to healthy cells depending on its concentration and light dose [11,12]. However, MB is prone to aggregate and undergo photobleaching in biological media [13,14]. In contrast, 1,9-dimethyl methylene blue (DMMB) is more lipophilic, more resistant to aggregation and produces a higher quantum yield of singlet oxygen (^1^O_2_) than that of MB [15]. Although it is able to mediate the PDI of azole-resistant *Candida albicans* in planktonic cells [16], its use on *C. auris* remains unexplored.

**Figure 1:**
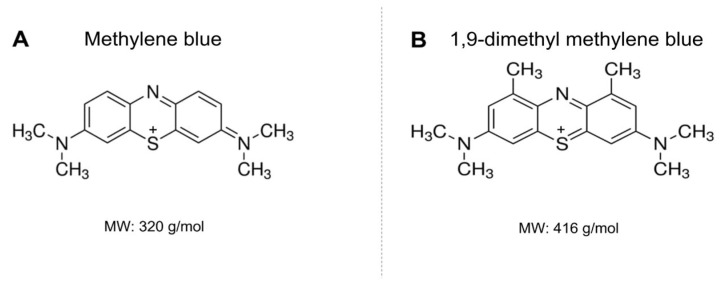
Chemical structures of MB (**A**) and DMMB (**B**). MW: molecular weight.

Here, we aimed to compare the effects of PDI mediated by MB (MB-PDI) and DMMB (DMMB-PDI) on *C. auris* planktonic cells (Figure 1). We obtained the response curve and calculated the number of absorbed photons for both PSs as well as obtained ROS kinetics for each PS following PDI. We also investigated the PS influence on lipid peroxidation (LPO) and mitochondrial membrane potential (ΔΨm). After, we decided to exploit the impact of DMMB-PDI on *C. auris* biofilms by verifying the metabolic activity and biomass. In this case, two approaches were used: (i-) the prevention of biofilm formation and (ii-) the rupture of mature biofilms. Biofilm morphological and structural changes were verified via scanning electron microscopy (SEM) and optical coherence tomography (OCT).

## 2. Materials and Methods

### 2.1. C. auris Growth Conditions

The *C. auris* CBS 10913 strain (first isolated from the auditory canal in Japan) [17], initially frozen at −80 °C, was thawed and cultured in Sabouraud Dextrose Broth (SDB, Himedia, Pennsylvania, USA) and incubated at 37 °C for 24 h. Subsequently, cells were seeded in Petri dishes containing Sabouraud Dextrose Agar (SDA, Kasvi, São José dos Pinhais, Brazil) for another 24 h at 37 °C. One colony of culture was dispersed in 2 mL phosphate-buffered saline (PBS). The yeasts were centrifuged (580× *g* for 5 min) and washed twice with PBS. Then, cells were resuspended to prepare the inoculum to a final concentration of ~1 × 10^7^ colony-forming units per milliliter (CFU/mL), adjusted using the optical density at 540 nm (OD540) in a spectrophotometer (SpectraMax M4, Molecular Devices, San Jose, CA, USA). The antifungal susceptibility of CBS 10913 has been recently reported, and the minimal inhibitory concentration (MIC) in mg/mL for some antifungals is 0.064 (Micafungin), 8 (Fluconazole), 0.125 (5-Flucytosine), 1 (Amphotericin B) and 0.032 (Ibrexafungerp) [18].

### 2.2. Photosensitizers and Light Source

Stock solutions of MB (1 mM) and DMMB (300 μM) (Sigma Aldrich, St Louis, MI, USA) were prepared in PBS and kept in the dark. MB and DMMB were used at different concentrations of 100, 50, 25 and 12.5 μM or 3, 1.5, 0.75 and 0.375 μM, respectively. An LED at λ = 662 (15) nm (BioLambda, São Paulo, Brazil) with an irradiance of 50 mW/cm^2^ was used to deliver light doses of 10 or 30 J/cm^2^ for 3 min 18 s and 9 min 56 s, respectively.

### 2.3. Cell Suspension Preparation and PDI

Initially, an inoculum with a concentration of 1 × 10^7^ CFU/mL, as previously described, was prepared in PBS. Sterile 96-well plates were used for each light dose. To perform PDI, each plate was inoculated with 100 μL of inoculum and 100 μL of MB or DMMB at different concentrations and then incubated in the dark at 37 °C with a pre-irradiation time (PIT) of 10 min to allow PS uptake. Then, the plates were irradiated at light doses of 10 or 30 J/cm^2^. Under the same conditions, MB or DMMB were incubated in the dark at 37 °C as a control without light. An untreated control (no PS and no light) and light groups (no PS, only light) were also used.

After irradiation, 10 μL aliquots from each group were collected, serially diluted (1:10), inoculated in Petri dishes and then incubated at 37 °C for 24 h. The number of colonies was determined as CFU/mL = (number of colonies on the dish) × (reverse plated dilution coefficient) × 10^2^ (contents seeded on the dish in 1 mL). At least 3 independent experiments were performed in triplicate.

### 2.4. Number of Absorbed Photons

To estimate the number of absorbed photons per second (Equation (1)), we used the emission spectra of the red LED normalized according to the LED peak emission [P(λ)] and the absorbance of DMMB (3 μM) and MB (100 μM) normalized with respect to the PS absorption peaks [A(λ)] to allow a proper comparison between PSs. PS absorbance was obtained with an optical length of 11 mm with a spectrometer (SpectraMax M4, Molecular Devices, San Jose, CA, USA) from 450 nm to 750 nm.

(1) $$\int_{\lambda_{0}}^{\lambda }\frac{P\left(\lambda \right).10^{A\left(\lambda \right)}}{c.h}d\lambda \;$$

where

c: velocity of light in vacuum (~3 × 10^8^ m/s);
h: Planck constant (6.62606957 × 10^−34^ J·s).

### 2.5. ROS Kinetics

To monitor ROS, we used an inoculum of 1 × 10^7^ CFU/mL of *C. auris* irradiated at light doses of 10 or 30 J/cm^2^. The light dose and PS concentrations (MB at 100 µM, DMMB at 3 µM) were determined according to the previous experiment. Immediately after PDI, each well of the plate was incubated with the permeant fluorogenic dye 2′,7′-dichlorofluorescein diacetate (DCFDA-DA, Sigma Aldrich, St Louis, MI, USA) at a final concentration of 10 μM. Dark groups were used as negative controls. The kinetics of ROS production was evaluated using fluorescence (λ_ex_: 485 nm and λ_em_: 538 nm) in a spectrophotometer at 37 °C (SpectraMax M4, Molecular Devices, San Jose, CA, USA) for 6 h. We also monitored ROS in the absence of cells and subtracted the values to plot ROS generated inside *C. auris*. To quantify ROS levels, we calculated the area under the curves.

### 2.6. Peroxidation Lipid and Mitochondrial Membrane Potential (ΔΨm)

To gain mechanistic insights into MB-PDI and DMMB-PDI on *C. auris*, it is necessary to use PDI sublethal conditions because complete killing does not allow for a proper analysis of death mechanisms. Thus, cell suspensions, under the conditions described in Section 2.3, were seeded into 96-well plates and treated with LC50 with the lethal conditions that killed 50% of cells (50 µM and 30 J/cm^2^ for MB and 0.75 µM and 10 J/cm^2^ for DMMB) to investigate LPO and ΔΨm. These parameters were selected based on two factors: (i-) the lowest light dose was not able to promote 50% killing for MB, and (ii-) at 0.75 μM, DMMB promoted 50% killing regardless of the light dose.

For the LPO assay, 10 µM of the Image-iT Lipid kit for live cells (Thermo Fisher, Waltham, MA, USA) was added to each well at 28 °C for 30 min and washed 3× with PBS. Images were captured with a fluorescence microscope (Nikon, Tokyo, Japan). The lipid peroxidation was quantified, as instructed by the manufacturer, by using the ImageJ^®^ software (ImageJ 1.53T, 64-bit), which provided the mean of the signal intensity for reduced (red emission) and oxidized (green emission) lipids of the microscopy images as a whole. We used 5 images per group.

Regarding ΔΨm, the cells were incubated with MitoTracker CMXRos (Invitrogen, Thermo Fisher, Waltham, MA, USA) at 28 °C in a final concentration of 100 nM for 45 min, immediately and 1 h after PDI. After that, cells were centrifuged at 580× *g* for 5 min and washed with PBS. Then, the fluorescence intensity was quantified by using a spectrophotometer (Spectramax M4, MolecularDevices, San Jose, CA, USA) at λ_ex_: 579 nm and λ_em_: 599 nm. For labeling representative cells, they were treated under the same conditions as above, followed by fixation with 2% paraformaldehyde for 20 min at room temperature. *C. auris* cells were allowed to attach to poly-L-lysine-coated slides before staining with 4,6-diamidino-2-phenylindole (DAPI, Sigma-Aldrich, St Louis, MI, USA) for 5 min at 2 µg/mL. Images were captured with a fluorescence microscope (Nikon, Tokyo, Japan). Data for LPO and ΔΨm were normalized concerning the untreated control group.

### 2.7. Biofilm Formation and PDI

To evaluate the effect of DMMB-PDI on biofilms, we used two approaches: (i-) the prevention of biofilm formation via growth inhibition and (ii-) the rupture of the mature formed biofilm. After 24 h of cultivation in SDB, *C. auris* cells were harvested (580× *g* for 5 min) and washed twice with PBS. Thereafter, the yeast suspension was diluted in RPMI 1640 (Gibco, Thermo Fisher Scientific, Waltham, MA, USA without phenol red, supplemented with 20 mM HEPES) to a final concentration of ~5 × 10^7^ CFU/mL using the OD540. A volume of 100 µL of *C. auris* was added to a 96-well plate and incubated at 37 °C for 90 min with constant rotation at 1× *g*. Two plates with biofilms (n = 5) were prepared for each test: one for the irradiated groups and the other for the dark groups.

For the first assay, fungal cells were carefully washed 2× after cell adhesion. The supernatant was removed, and 200 µL of PBS was added to remove non-adhered cells. Then, PBS was removed, and 200 µL of DMMB (3 µM) was added. After a PIT of 10 min, the plate was irradiated at 10 J/cm^2^. After irradiation, cells were gently washed twice with 200 µL of PBS and replenished with 200 µL of RPMI 1640. The plates were sealed and incubated at 37 °C for 48 h to allow biofilm formation and maturation.

The second assay was performed after the cell adhesion step, without previous irradiation. The wells were gently washed as previously reported, and plates were sealed and stored at 37 °C for 48 h for the growth and maturation of the biofilm. After 48 h, the plates were submitted to PDI under the same experimental conditions mentioned above.

### 2.8. Analysis of Metabolic Activity and Biomass in Biofilms

The effect of DMMB-PDI on metabolic activity in biofilms was evaluated via the 3-(4,5-dimethylthiazol-2-yl)-2,5-diphenyltetrazolium bromide (MTT) assay. Immediately after PDI, the wells containing *C. auris* biofilms were thoroughly washed twice with 200 µL of PBS, followed by the addition of 180 µL of RPMI 1640 and 20 µL of MTT (5 mg/mL, Sigma Aldrich, St Louis, MI, USA). The plates were then incubated in the dark for 4 h at 37 °C. The supernatant was removed from each well, and 200 µL of dimethyl sulfoxide (DMSO) was added. Then, the plates were kept in the dark under gentle shaking for 15 min to allow the formazan crystals to dissolve. After complete homogenization, 100 µL of the system was transferred to a new flat-bottomed 96-well plate for absorbance reading at 570 nm (SpectraMax M4, Molecular Devices, San Jose, CA, USA).

To evaluate *C. auris* biomass after DMMB-PDI, crystal violet (CV) staining (Synth, Diadema, Brazil) was used. Immediately after PDI, each well of the plate was washed gently with PBS, fixed in methanol for 20 min and dried in an oven at 37 °C. The samples were then stained for 15 min with 0.05% (*w*/*v*) CV. The CV solution was removed, and the samples were washed gently in distilled water. Finally, ethanol was used to dissolve the bound CV for 30 min, and the absorbance was measured at 492 nm using a microplate reader (SpectraMax M4, Molecular Devices, San Jose, CA, USA). Data were normalized concerning the untreated control group.

### 2.9. Structural Changes by SEM and OCT

Biofilms were grown in QX-102 wet sample capsules. To properly support the capsules, an MP-10 plate covered with RPMI 1640 was used. The methodology was the same as that reported in Section 2.5. Samples were analyzed via low-vacuum scanning electronic microscopy (TM 3000, Hitachi, Tokyo, Japan).

Regarding OCT, we used a device with λ = 1300 nm and an axial resolution of 10.6 µm (VEG220, Thorlabs, Newton, MA, USA). Two-dimensional and three-dimensional images of biofilms were constructed. Biofilm thickness was measured using two-dimensional images through ImageJ^®^ software (ImageJ 1.53T, 64-bit) for each experimental group. At least three independent experiments were performed.

### 2.10. Cytotoxicity on Mammalian Cells

NIH/3T3 mouse embryonic fibroblast cells were cultured in DMEM medium (15 mM HEPES, 2 g of sodium bicarbonate/L, and 1 mM L-glutamine) and supplemented with 20% fetal bovine serum (FBS) until reaching 70% confluence at an atmosphere of 5% of CO_2_ and 37 °C.

For this assay, 5 × 10^3^ fibroblasts were seeded on 96-well plates 24 h before the experiments. MB or DMMB was incubated for 10 min using the highest concentrations (100 and 3 μM, respectively). Then, cells were irradiated using the red LED delivering 30 J/cm^2^. Untreated cells were used as a negative control in a different plate. Light and PS groups were also assessed.

Following PDI, each well received 10 µL of resazurin (1.1 mg/mL, Sigma Aldrich, St Louis, MI, USA). The cells were then incubated again in a humid atmosphere containing 5% CO_2_ at 37 °C. After 4 h, the fluorescence was measured with a microplate reader with λ_ex_: 560 nm and λ_em_: 590 nm (SpectraMax M4, Molecular Devices, San Jose, CA, USA).

### 2.11. Statistical Analysis

All statistical analyses were evaluated using GraphPad Prism 7.0 software via one-way analysis of variance (ANOVA) followed by the Tukey post hoc test. Differences were considered statistically significant when *p* < 0.05. Data are plotted as mean values ± standard deviation (SD).

## 3. Results

**Figure 2:**
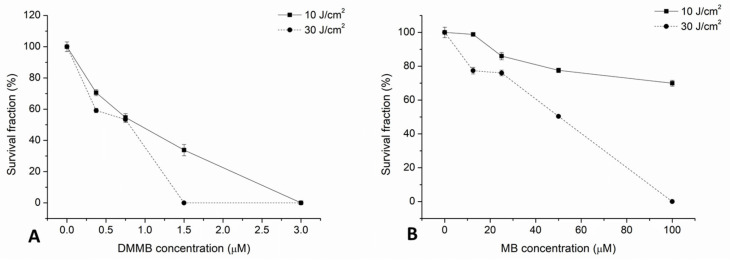
Response curves of *C. auris* planktonic cells exposed to 10 and 30 J/cm^2^ (red LED at 50 mW/cm^2^) for growing concentrations of DMMB (**A**) and MB (**B**).

Figure 2 exhibits the response curves for the planktonic cells of *C. auris*. Regardless of the PS, we observed that fungal killing depended on the PS concentration and light dose. For DMMB, the highest concentration (3 µM) resulted in the complete eradication of *C. auris* regardless of the light dose (Figure 2A). Additionally, the highest light dose was able to completely eradicate the fungal burden, even at 1.5 µM. Interestingly, low concentrations of DMMB (0.75 and 0.375 µM) showed similar killing regardless of the light dose.

On the other hand, although MB presented antifungal activity against *C. auris* for the highest light dose, complete eradication was only noticed at 100 µM (Figure 2B). In contrast, the lowest light dose was not able to kill 50% of cells even at the highest concentration of MB. For both PSs, the dark and light groups were similar to the untreated controls.

**Figure 3:**
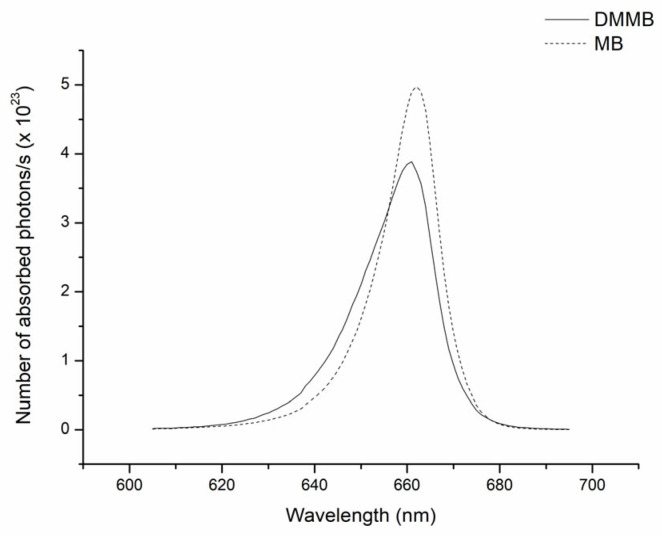
Number of absorbed photons/s for DMMB (3 μM) and MB (100 μM) for the red LED used in this study, λ = 662(15) nm.

Our results indicate that DMMB was more effective than MB at killing *C. auris*. However, DMMB was used in concentrations much lower than those of MB. Thus, we calculated the number of photons absorbed per second for each PS (Figure 3). Interestingly, by calculating the area under the curves presented in Figure 3, we determined that MB can absorb approximately 5% more photons/s than DMMB.

**Figure 4:**
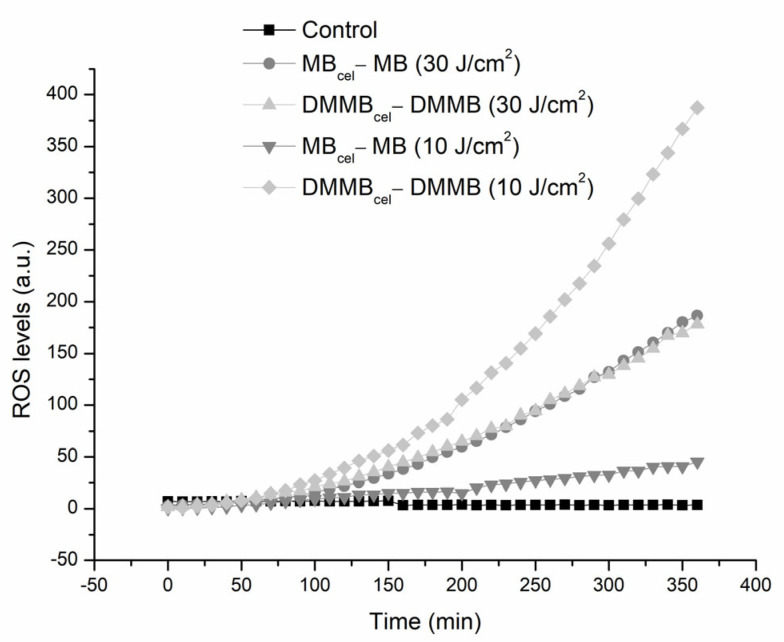
ROS generated inside *C. auris* cells over 6 h following DMMB and MB-PDI at 10 or 30 J/cm^2^.

The amount of ROS generated inside *C. auris* cells is presented in Figure 4. Comparing DMMB and MB, it is possible to observe that ROS levels for DMMB were approximately 92% higher than those for MB for 10 J/cm^2^. In contrast, similar levels were noticed for MB and DMMB at 30 J/cm^2^. Curiously, the quantity of ROS increased by around 70% for MB from 10 to 30 J/cm^2^. In contrast, ROS levels were reduced by approximately 55% from the lowest to the highest light dose for DMMB.

**Figure 5:**
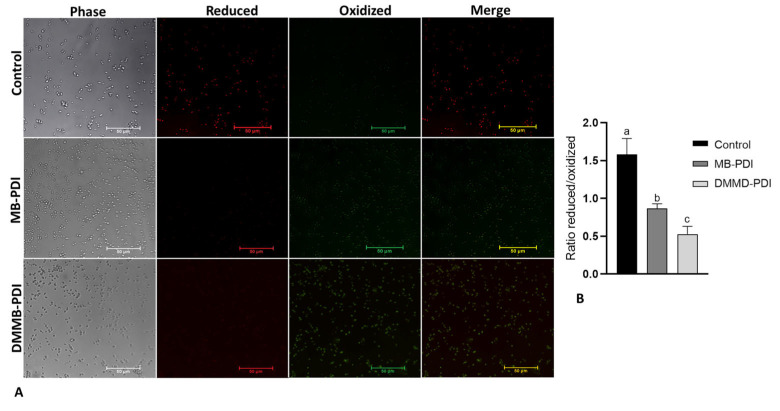
Representative photomicrographs of lipid peroxidation in *C. auris* planktonic cells following MB (50 µM, 30 J/cm^2^) and DMMB-PDI (0.75 µM, 10 J/cm^2^). Scale bar: 50 μm (**A**); Mean values ± SD of the reduced/oxidized ratio (**B**). Different letters represent statistically significant differences among groups (*p* < 0.05).

Regarding LPO, we observed that control cells showed a higher fluorescence signal in the reduced channel, as expected (Figure 5A). On the other hand, the PDI groups showed a higher signal in the oxidized channel, especially DMMB-PDI (Figure 5A). Indeed, the reduced/oxidized ratio was significantly lower for DMMB than that for MB (around 40%) (Figure 5B).

**Figure 6:**
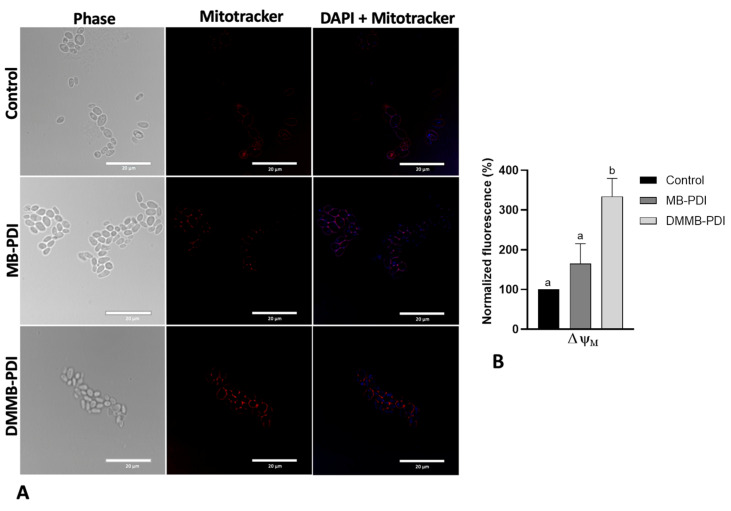
Representative photomicrographs of mitochondrial membrane potential in *C. auris* planktonic cells following MB (50 µM, 30 J/cm^2^) and DMMB-PDI (0.75 µM, 10 J/cm^2^). Nuclei were stained with DAPI (blue fluorescence), and mitochondria were stained with MitoTracker red (red fluorescence). Scale bar: 20 µm (**A**); Mean values ± SD of the normalized fluorescence (**B**). Different letters represent statistically significant differences among groups (*p* < 0.05).

**Figure 7:**
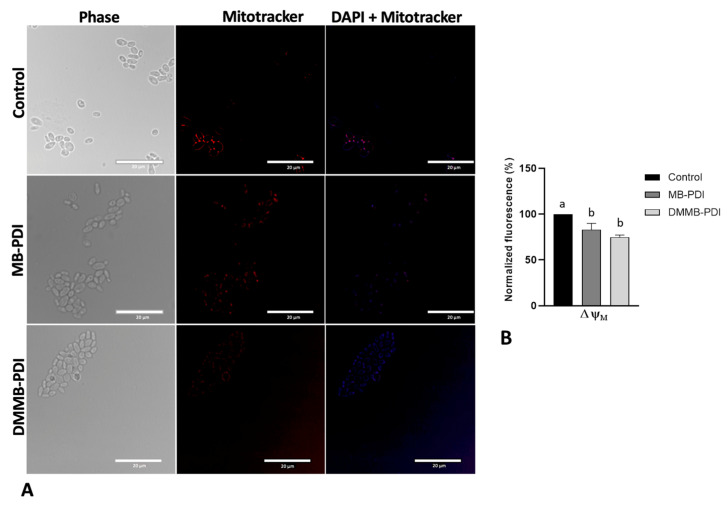
Representative photomicrographs of mitochondrial membrane potential in *C. auris* planktonic cells after 1 h of MB (50 µM, 30 J/cm^2^) and DMMB-PDI (0.75 µM, 10 J/cm^2^). Nuclei were stained with DAPI (blue fluorescence), and mitochondria were stained with MitoTracker red (red fluorescence). Scale bar: 20 µm (**A**); Mean values ± SD of the normalized fluorescence (**B**). Different letters represent statistically significant differences among groups (*p* < 0.05).

We also evaluated the ΔΨm immediately and 1 h after PDI (Figure 6 and Figure 7). Following PDI, we observed a higher polarization signal for MB than that of the control, even though no statistically significant differences were detected. In contrast, DMMB-PDI promoted a significant hyperpolarization of the mitochondrial membrane, approximately two-fold higher than that of MB (Figure 6). Interestingly, after 1 h, both MB and DMMB-PDI groups exhibited a significant loss of ΔΨm, indicating depolarization when compared to the untreated control group (Figure 7A). However, no statistically significant differences were noticed between the MB and DMMB-PDI groups (Figure 7B).

**Figure 8:**
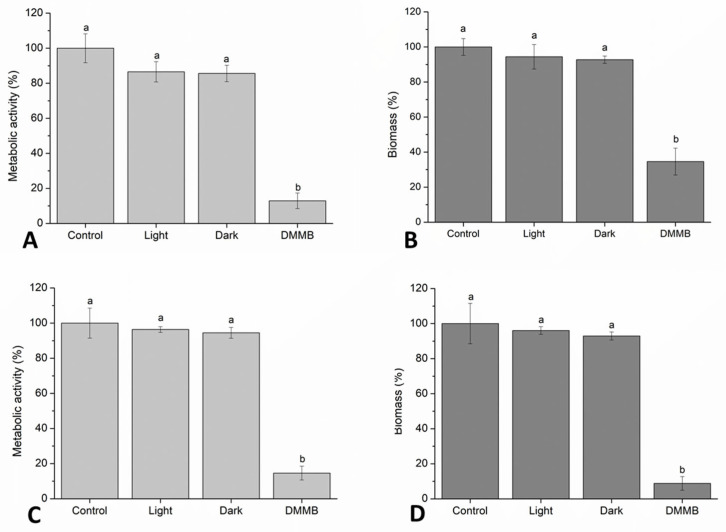
Mean values ± SD of the mitochondrial activity and biomass for growth inhibition (**A**,**B**), and biofilm disruption (**C**,**D**) following DMMB-PDI (3 μM and 10 J/cm^2^). Different letters represent statistically significant differences (*p* < 0.05).

As DMMB-PDI showed better results for planktonic cells, we decided to investigate its potential to prevent biofilm formation and to disrupt mature biofilms (Figure 8). We observed that DMMB-PDI inhibited biofilm formation by 87% of the metabolic activity and 65% of the biomass when compared to the untreated controls (Figure 8A,B). For mature biofilms, the metabolic activity and biomass were reduced by 85% and 91%, respectively (Figure 8C,D). Dark and light groups showed similar values to those of the untreated controls.

**Figure 9:**
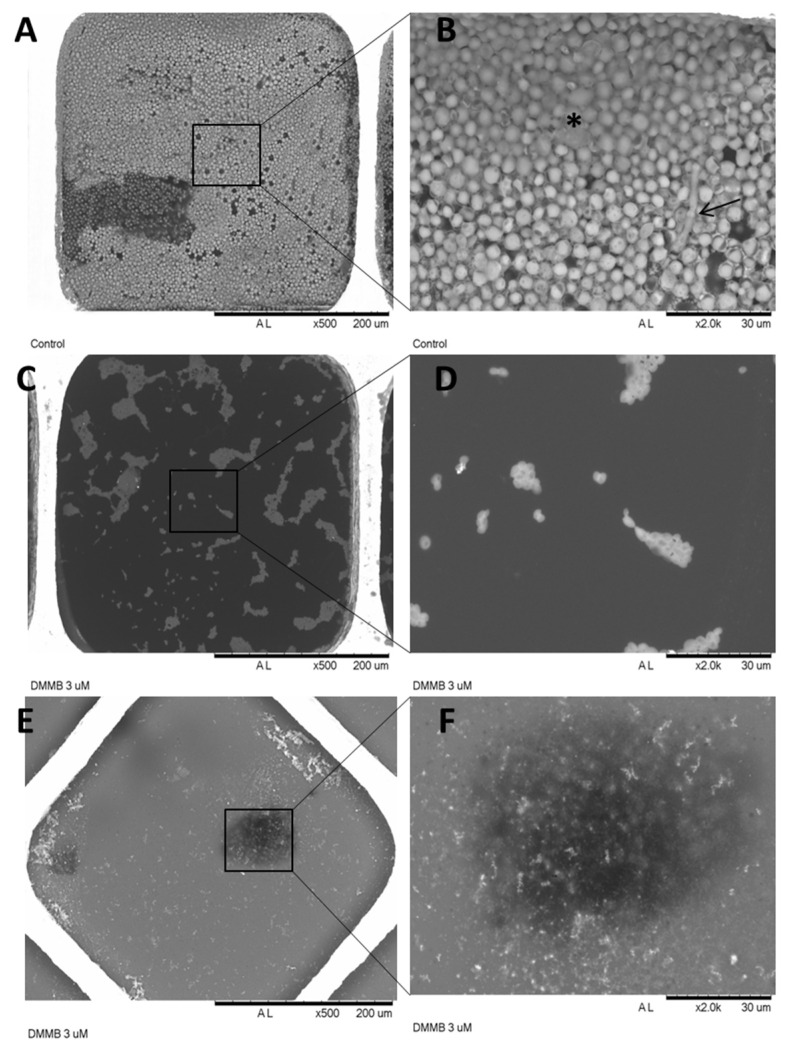
Representative scanning electron micrographs of *C. auris* biofilms for untreated control (**A**,**B**), DMMB-PDI inhibiting biofilm formation (**C**,**D**) and rupture of mature biofilm by DMMB-PDI (**E**,**F**). DMMB was used at 3 μM, and the light dose was 10 J/cm^2^. * denotes extracellular polymeric substances, and the arrow points to hyphae.

Morphological changes promoted by DMMB-PDI can be observed in Figure 9. The biofilms formed by *C. auris* consisted of a robust homogeneous structure of yeast-shaped cells embedded in extracellular polymeric substances. Hyphae were also observed (Figure 9A,B). Following DMMB-PDI to prevent biofilm formation, we noted the formation of a few multicellular aggregates without the apparent complexity of a biofilm (Figure 9C,D). Furthermore, DMMB-PDI drastically disrupted the mature biofilm because no whole yeasts were perceived (Figure 9E,F).

**Figure 10:**
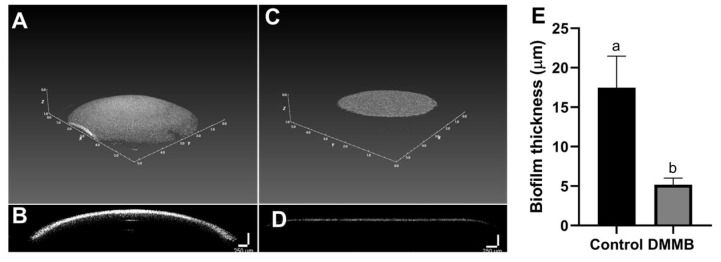
*C. auris* biofilm profile for the growth inhibition assay evaluated with OCT. Untreated control biofilm (**A**,**B**) and DMMB-PDI (3 µM, 10 J/cm^2^) (**C**,**D**). (**A**,**C**) show 3D biofilm representative images; (**B**,**D**) show the cross-section of the biofilm. Biofilm thickness is shown in (**E**). Different letters represent statistically significant differences (*p* < 0.05). Similar biofilm images and thicknesses were observed for the biofilm rupture assay.

The OCT allowed for the analysis of the thickness of biofilms and allowed us to perform a 3D reconstruction of these structures (Figure 10). For the growth inhibition assays, untreated biofilms showed a thickness of about 17 µm. After DMMB-PDI, biofilm thickness was significantly reduced by approximately 71% (~5 µm) (Figure 10E). The 3D reconstruction revealed the pronounced effect on the spatial shape of biofilms treated with DMMB-PDI, which exhibited a smaller volume compared to that of the control (Figure 10A–D). Biofilm thicknesses were rather similar in the biofilm rupture assay.

**Figure 11:**
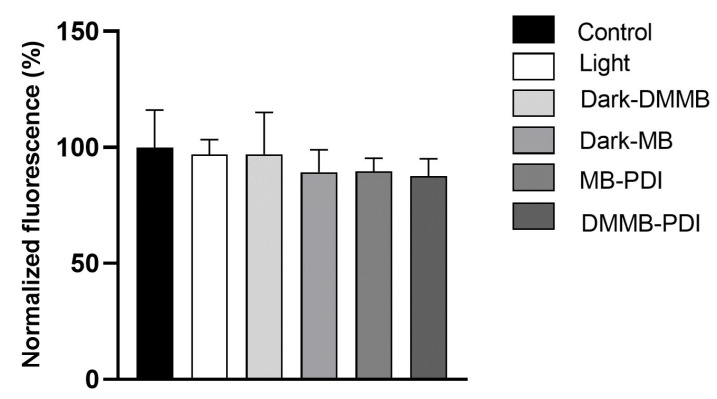
Mean values ± SD of the cell viability of murine fibroblasts submitted to different treatments. Cells were incubated with the PS for 10 min before irradiation. MB was used at 100 μM, and DMMB was used at 3 μM. The light dose was 30 J/cm^2^.

As DMMB is less explored in vivo, we checked DMMB toxicity on fibroblasts. Here, MB was used as a positive control because it seems to be safe in clinical practice. No significant toxicity was observed. Dark and light groups were also nontoxic to fibroblasts. All groups showed similar viability to that of the untreated control cells (Figure 11).

## 4. Discussion

MB is a well-recognized PS used in antifungal PDI. In vitro, studies have reported its potential to kill different fungal species [19,20] in addition to treating infectious diseases such as candidiasis and onychomycosis [21,22]. DMMB, on the other hand, is produced from the methylation of MB. Although it has been used as a metachromatic stain, its use in PDI to kill fungi remains unexplored. Table 1 presents our data and those from other works that have reported MB-PDI or DMMB-PDI against *Candida* spp. Although PS concentrations and light parameters are rather dissimilar among studies, we observed that (i-) PDI is equally effective for antifungal-resistant and susceptible *Candida* spp.; (ii-) DMMB is used in much lower concentrations than those for MB; and (iii-) the PDI protocol impacts the killing of *Candida* cells. Additionally, DMMB seems to be more effective at killing *C. auris* because we obtained 100% killing for suspension cells.

Regarding our data, we noticed some particularities for response curves (see Figure 2). In the lowest light dose, MB at 100 μM was not able to kill more than 45% of cells. In contrast, DMMB at 3 μM was able to promote the complete eradication of *C. auris* regardless of the light dose.

MB and DMMB are phenothiazines that absorb red light but that have absorption peaks that are slightly different (656 and 650 nm, respectively) [15]. Thus, we calculated the number of photons absorbed per second, which allows a comparison of the photodynamic effect per excited molecule of each PS [26]. Although MB absorbs more photons than DMMB, DMMB could generate more ROS than MB for the lowest light dose (10 J/cm^2^), which supports our findings. However, MB and DMMB showed similar levels of ROS for the highest light dose (30 J/cm^2^), which was enough to completely eradicate *C. auris* yeasts.

These data can be explained by the characteristics of PS and *C. auris* antioxidant defenses. Both PSs are cationic molecules (i.e., positively charged) engaged in both type I and II photodynamic reactions. However, the quantum yield of ^1^O_2_ for DMMB is around 30% higher than that for MB (0.71 vs. 0.51, respectively) [27]. Additionally, the antioxidant defense mechanism for fungi, including *Candida albicans*, comprises the induction of antioxidant-encoding genes of catalase, superoxide dismutase and glutathione peroxidase, as well as genes encoding constituents of the glutathione/glutaredoxin and thioredoxin systems [28,29]. This antioxidant system could fight mainly the superoxide anion and hydrogen peroxide, both generated by a type I reaction. On the other hand, ^1^O_2_ is mainly extinguished by carotenoids, which are pigments that hold triplet energy levels close to that of ^1^O_2_, allowing energy transfer between both molecules and ^1^O_2_ quenching [30]. Fungi accumulate low levels of carotenoids [31], so *Candida utilis*, an industrially important yeast, does not synthesize carotenoids [32]. Because our assay did not distinguish different ROS and there is not enough information about the antioxidant mechanisms of *C. auris*, we assumed that the cells were more susceptible to ^1^O_2_ generated by the photoactivation of DMMB when we used 10 J/cm^2^. For the highest dose, the fungal antioxidant defenses were likely depleted because MB and DMMB were equally effective.

Our data also show that LPO was more pronounced following DMMB-PDI. DMMB and MB differ regarding their efficacy in interacting and damaging membranes. DMMB is more lipophilic than MB (partition coefficient of +1.01 and −0.1, respectively), which enables a more efficient bond to the cell membrane and ^1^O_2_ lifetimes that are two times higher (around 4.1 vs. 2.2 μs, respectively) [27]. Hence, ^1^O_2_ is highly reactive against unsaturated lipids, triggering lipid peroxidation by hydrogen abstraction [33].

Due to their positive charge, DMMB and MB are prone to accumulate in mitochondria, which have a negative ΔΨm [34]. Immediately after PDI, although both PSs were able to hyperpolarize the mitochondrial membrane, a higher polarization was observed for DMMB. However, 1 h after PDI, ΔΨm was lower than that of the control, indicating membrane depolarization for both PSs. These findings indicate that the increase in ΔΨm is transitory because excessive ROS formation would lead to a loss of ΔΨm and mitochondria destruction [35]. However, other authors have also observed ΔΨm hyperpolarization following photodynamic treatment [36,37]. We hypothesize that PDI-induced mitochondrial permeability transition pores (PTPs) may cause swelling and other structural changes triggering cell death [38]. Indeed, the opening of PTPs results in a brief hyperpolarization phase, which corresponds to the onset of ROS production [39].

In front of this outcome, we decided to verify the effects of DMMB-PDI on the formation and rupture of biofilms. As with other *Candida* spp., the complex community of the *C. auris* biofilm is associated with drug resistance, virulence and survival [40]. *C. auris* biofilms are formed by aggregated or non-aggregated yeast-like cells with sporadic pseudo hyphae that are surrounded by an extracellular matrix that is rich in polysaccharides [41,42], which confirms our findings. Biofilms can colonize and grow on surfaces, such as the mucosa and catheter, which can disseminate to other foci, causing candidemia when reaching the bloodstream. *C. auris* can contaminate and survive on environmental surfaces for more than 3 weeks [43]. In this context, this fungus demands new efforts to prevent and treat infections.

MB-PDI has already been shown to be effective against the *C. auris* biofilm formed by 24 h at a concentration of 250 μM under a light dose of 57 J/cm^2^ [8]. Here, we found that DMMB-PDI was able to prevent the formation of biofilms in addition to destroying structures that were mature after 48 h at a much lower concentration (3 μM) and a light dose of 10 J/cm^2^. For both assessed conditions, DMMB-PDI significantly reduced metabolic activity and biomass. Morphological changes involved the inhibition of biofilm growth and the destruction of mature biofilms. The thickness of biofilms drastically dropped following DMMB-PDI, which is in line with our biomass data. Taken together, DMMB-PDI could be a new weapon to fight *C. auris* infections.

Last but not least, we verified the cytotoxicity of DMMB-PDI on fibroblast cells. No toxicity was noticed under the tested conditions, indicating that DMMB-PDI inactivates *C. auris* rather than normal cells, so it could be safe in clinical trials.

## 5. Conclusions

Given the global threat posed by *C. auris*, including its resistance to conventional drugs and its ability to adhere to and to form biofilms, there is a growing need for alternative antifungal approaches. The present study demonstrates a promising approach to eradicate *C. auris* by using DMMB-mediated PDI. More importantly, DMMB-PDI was effective in inhibiting biofilm formation and causing biofilm destruction. These findings open an avenue to explore its use in surface disinfection and to investigate its in vivo potential.

## Figures and Tables

**Table 1:** Data from some works for MB-PDI and DMMB-PDI on *Candida* spp. NI: not informed; *: PS concentration in μg/mL converted to μM.

*Candida* Strain	Form	PS (Concentration) (µM)	PIT (min)	Light Source	λ_max_ (nm)	Irradiance (mW/cm^2^)	Light Dose (J/cm^2^)	Exposure Time (s)	Outcome
*C. albicans* (ATCC 18804 and Ca70 fluconazole-resistant)	Planktonic	MB (300)	15	LED	660	42	30	714	~99% log reduction regardless of the resistance pattern
	Biofilm 48 h	MB (600)							~99.9% reduction regardless of the resistance pattern [23]
*C. albicans* ATCC 10231, *C. parapsilosis* ATCC 22019 and *C. krusei* ATCC 6258	Planktonic	MB (1000) *	0	LED	625	7	18	2571.6	99.9999% reduction regardless of the strain. PS concentration for *C. parapsilosis* was 250 μM [24]
				White lamp	420–700	90		205	
*C. auris* (H261 and fluconazole-resistant)	Biofilm 24 h	MB (250)	15	Laser	660	190	58	300	>90% reduction [8]
*C. albicans* (ATCC 10231 and AZN9635, 456325H, AMO7/0267 azole-resistants)	Planktonic	DMMB (0.6 to 2.5)	NI	LED	639.8	19	18 and 37	NI	>99.9% reduction regardless of the resistance pattern [16]
*C. albicans* (ATCC 90028)	Planktonic	DMMB (2.4) *	5	LED	630	41.2	20	870	99.9% reduction [25]
*C. auris* (CBS 10913)	Planktonic	MB (100 μM) DMMB (1.5 μM)	5	LED	662	50	30	596	100% reduction
	Biofilm 24 h	DMMB (3 μM)					10	198	>85% reduction (this work)

## Data Availability

Data are available upon reasonable request.
